# 

**Published:** 1916-10-21

**Authors:** 


### CONTENTS.

| | Page |
|---|---|
| **A Notable Contest** ... ... ... ... ... ... | 37 |
| **The Great Extension at Leeds** ... ... ... | 38 |
| **Hospital and Institutional News** ... ... ... | 39 |
| The Empress Eugénie's Hospital for Wounded Officers—Opening of the New Russian Hospital—The Small Military Hospital—Cavell Memorial at Shoreditch Infirmary—The Scandal of the Indian Hospitals—Hospital Finance in New Zealand—The Grove Hospital for Wounded Soldiers—The New Chairman of Addenbrooke's Hospital—A Point for the President of the L.G.B.—Sphagnum Dressings in Hampshire and Berkshire—Munition Workers and T.N.T. Poisoning—Petty Patronage in its Last Ditch—The Initials on the Wall—Human Temperament—A Belated Appeal—The Great Northern Central Hospital—The Bishop and the Wounded—This Week's Drug Market. | |
| **The American Hospital Association** ... ... | 43 |
| Its Progress and Present Position. | |
| **Hospital and Institutional Results in 1915** ... | 44 |
| **Œdema and Anasarca**... ... ... ... ... | 45 |
| Some Overlooked Factors in Causation. | |
| **The Future of School Medical Inspection** ... | 46 |
| By a School Medical Officer. | |
| **Life and Death: Some Human Lessons in War-Time** ... ... ... ... ... ... | 47 |
| V.—The Pleasures of Life. | |
| **Human Temperaments** ... ... ... ... | 49 |
| XIII.—The Suspicious Temperament. | |
| **National Insurance Act Developments** ... ... | 51 |
| Discharged Soldiers' Allowances. | |
| **Some Military Hospital Gazettes** ... ... ... | 52 |
| **The Matrons' and Sisters' Department** ... | 53 |
| Round the Hospitals. | |
| More About the Scottish Board—A New Massage Institute—Lectures to Tired Probationers—Shortage of Trained Nurses—A Matron and Patrol Work—Imperial Nurses' Club. | |
| **Patriotism and Nurse-Training Schools** ... | 54 |
| IX.—Royal Infirmary, Edinburgh. | |
| **The Editor's Letter-Box** ... ... ... ... | 55 |
| Mental Nurses in War Hospitals. | |
| **Matrons' and Sisters' Appointments** ... ... | 56 |
| **Parliament and the Profession** ... ... ... | 56 |
| **Obituary** ... ... ... ... ... ... ... ... | 56 |
| **The Institutional Worker** ... ... (Suppl.) | 1 |
| Accuracy in Medicine Giving. | |
| Question for October. | |
| Enquiries and Answers. | |
| The Sick and in Need. | |
| Employment and Training. | |
| Practical Matters. | |

